# On the Way to Mars—Flagellated Algae in Bioregenerative Life Support Systems Under Microgravity Conditions

**DOI:** 10.3389/fpls.2019.01621

**Published:** 2020-01-08

**Authors:** Donat‑P. Häder

**Affiliations:** Department of Biology, Friedrich-Alexander-Universität Erlangen-Nürnberg, Erlangen, Germany

**Keywords:** *Euglena gracilis*, flagellate, bioregenerative life support system, microgravity, oxygen, carbon dioxide

## Abstract

For long-term manned interplanetary missions it is not feasible to carry the necessary oxygen, food, and water to sustain the astronauts. In addition, the CO_2_ exhaled by the astronauts has to be removed from the cabin air. One alternative is to utilize photosynthetic organisms to uptake the CO_2_ and produce oxygen. In addition to higher plants, algae are perfect candidates for this purpose. They also serve to absorb wastes and clean the water. Cyanobacteria can be utilized as food supplement. Early ground-based systems include micro-ecological life support system alternative, closed equilibrated biological aquatic system, and the Biomass Production Chamber. The AQUARACK used the unicellular flagellate *Euglena* which produced the oxygen for fish in a connected compartment. A number of bioregenerative systems (AQUACELLS, OMEGAHAB) have been built for experiments on satellites. A later experiment was based on a 60-ml closed aquatic ecosystem launched on the Shenzhou 8 spacecraft containing several algae and a small snail living in adjacent chambers. Recently the Eu : CROPIS mission has been launched in a small satellite within a Deutschen Zentrum für Luft- und Raumfahrt (DLR) program. In addition to tomato plants, *Euglena* is included as oxygen producer. One new approach is to recycle urine on a bacterial filter to produce nitrogen fertilizer to grow vegetables.

## Introduction

If humans should ever decide to send astronauts to Mars or other distant celestial bodies, the mission will pose a number of difficult problems. In addition to challenging technological tasks and microgravity- and radiation-related health issues, the crew needs to carry food and oxygen for a long-term interplanetary travel lasting months or years (Merrill et al., 2015; Lazendic-Galloway and Overton, 2017). The exhaled CO_2_ has to be removed from the cabin air (Rogers et al., 2017; Kamire and Yates, 2018) while water can be recycled as is already been done on the International Space Station (Moores et al., 2015; Ritter, 2018). On the International Space Station (ISS) all water (graywater, sweat, moisture in breath and urine) is collected in a closed-loop recycling system where impurities and contaminants are filtered out of the water.

Since these requirements for humans are too big a burden for current technological possibilities, alternatives have been considered during the last decades. One important approach was to engage bioregenerative life support systems to recycle at least the gases. For this purpose photosynthetic organisms are ideal components since they absorb the exhaled CO_2_ from the cabin air and produce oxygen as a side product. Algae are promising organisms to support humans during long space travel because they can recycle waste, remove carbon dioxide, and provide oxygen. However, green algae induced digestive problems even at small doses in the diet while cyanobacteria were found digestible (Escobar and Nabity, 2017) (https://ttu-ir.tdl.org/bitstream/handle/2346/73083/ICES_2017_311.pdf?sequence=1&isAllowed=y).

### Ground-Based Bioregenerative Life Support Systems

MELISSA (micro-ecological life support system alternative) has been developed to grow vegetables on the MIR station. In addition to a compartment for higher plants, it has a gas-lift photobioreactor for *Spirulina platensis* (Hendrickx et al., 2006). *Spirulina* is a photosynthetic cyanobacterium. It is edible and the Aztecs as well as some African tribes are reported to use it as human food. The organisms contains 55–70% protein, 15–25% carbohydrates, 18% essential fatty acids in addition to vitamins, minerals, and pigments. CEBAS (closed equilibrated biological aquatic system) was another development to test various organisms for their suitability in life support systems in space. The system has been tested with a 150-L container for more than 13 months (Bluem and Paris, 2001). Later an 8.5-L minimodule was built which contained the hornweed *Ceratophyllum demersum* as oxygen producer, the green swordtail (*Xiphophorus helleri*), and the air-breathing freshwater snail (*Biomphalaria glabrata*) as consumers (Blüm, 2003). This system was tested during two space shuttle missions (STS-89 and STS-90). In another experiment a small edible aquatic plant (*Wolffia arrhiza*) was used (Bluem and Paris, 2001).

The NASA Biomass Production Chamber (BPC) has been built to produce higher plants such as wheat, soybean, lettuce, and potato on a 20 m^2^ area (Wheeler et al., 1996). This system has been running for over 1,200 days without major problems. Likewise a bioregenerative life support system (Bios-3) has been developed in Krasnoyarsk, Siberia which uses higher plants or green algae to remove carbon dioxide, produce oxygen and even food (Salisbury et al., 1997). The largest system devised as closed bioregenerative life support system is biosphere 2 located near Oracle, Arizona. It is a large glass dome (480 m^3^) which harbored diverse biomes, such as ocean, lower thornscrub, and savannah and housed eight crew members for 2 years (1991–1993) (Nelson et al., 1993). The design concept was to create an independent ecosystem with no material transport from or to the outside but with energy input from the outside. However, this experiment was not successful because of oxygen depletion. The usage of plants in space-flown bioregenerative life support systems generates a number of problems. Any portion of the plant that is not part of the astronaut’s diet such as stems, roots, etc. must be treated as waste (Gitelson et al., 2003). This is also true for algae, although the algae could be part of a fish diet, and the fish be part of the human diet (Taub, 1963).

An early concept to study long-term cultivation in an algal bioreactor was proposed by Häder and Kreuzberg (1990). This design used an external CO_2_ and fresh media supply. Important physical and chemical parameters were measured on-line with inserted electrodes. At regular intervals part of the cell suspension was pumped through a closed loop with an observation cuvette in which the motility and orientation of the cells could be monitored using automatic cell tracking.

The next developmental step was based on the concept of a completely closed system (AQUARACK) without external supply of nutrients and CO_2_ (Lebert and Häder, 1998). Also in this system important chemical and physical parameters were constantly monitored and the motility and orientation analyzed at regular intervals. This system was used for long-term cultivation of the unicellular freshwater flagellate *Euglena gracilis* Ehrenberg for more than 600 days in an 11-L tank (Porst et al., 1997) (https://www.researchgate.net/publication/11803867_Long-term_cultivation_of_the_flagellate_Euglena_gracilis). This flagellate is about 80–120 µm long and possesses a single apical flagellum. It uses light (phototaxis) and gravity (gravitaxis) to orient itself in its microenvironment (Häder and Hemmersbach, 2017; Häder and Iseki, 2017). In the absence of light the cells can also grow heterotrophically on various organic media including sugars and amino acids. Because of its easy growth conditions, the organisms are often used in school experiments. The flagellate is not toxic, it is consumed by invertebrates such as *Daphnia* and fish since it is rich in protein and has a high nutritional value. The organisms are also grown as a food source for humans in the form of health supplements and drinks (https://www.runsociety.com/food-nutrition/euglena-a-superfood-with-powerful-benefits/). Further advantages are that the flagellates can be stored at room temperature and ambient light as a unialgal stock culture for years. The long-term culture experiment described above showed that the flagellates were not overgrown by other microbes. Large-scale cultivation of *Euglena* is described in a recent review (Suzuki, 2017).

In addition to monitoring oxygen concentration as well as motility and orientation, absorption spectra were measured to determine the chlorophyll and carotenoid content of the cells. During about a month a second aquarium was connected holding 15 snails (*B. glabra*) and 4 fish (*X. helleri*) to substitute for human consumers. The fish were fed automatically to sustain their growth (Strauch et al., 2008). The water with the oxygen produced by *Euglena* under external irradiation was passed to the zoological compartment *via* a membrane exchanger, which also allowed transporting the CO_2_ and waste products in the opposite direction to support photosynthesis and growth of the algae. The cell density increased over the first 150 days and then dropped to almost the initial value. At the end of the experiment the cell density sharply declined. Motility and velocity were almost constant over the duration of the experiment while gravitaxis steadily declined over time. The latter effect could be explained since the specific cell density decreased over time (as measured after the end of the long-term cultivation) so that it was not sufficient to exert enough pressure on the membrane-sensitive ion channels responsible for mechanoperception (Häder et al., 2009). The oxygen production was sufficient to support the animals in the zoological compartment. A closed loop photobioreactor for algae (*Chlorella vulgaris*) biomass production was tested on parabolic flights (Bretschneider et al., 2016). This system is part of the DLR photobioreactor in the life support rack designed to be installed on board the ISS in the Destiny module. It combines a bioreactor housing algae as oxygen producers with a carbon dioxide concentrator (Keppler et al., 2018).

### Bioregenerative Life Support Systems in Earth Orbit

After these initial terrestrial closed systems a number of bioregenerative systems (AQUACELLS, OMEGAHAB) have been built for space missions mounted on Russian Foton satellites.

**Figure 1:**
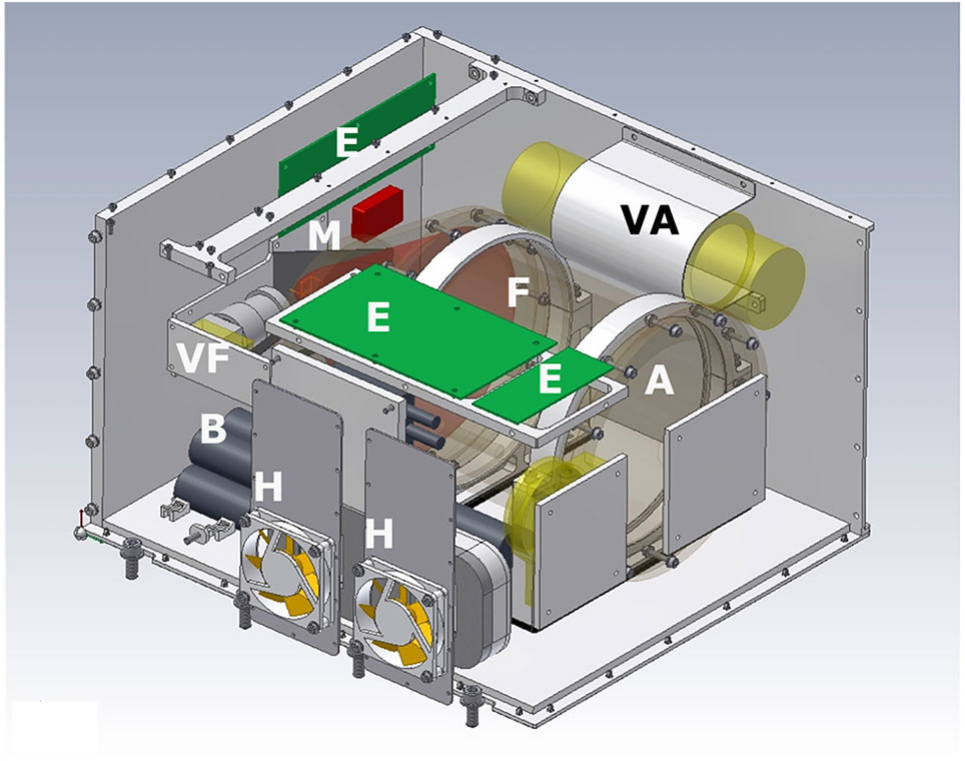
Schematic view of the Aquacells hardware showing the algal (A) and fish (F) tanks (combined in one transparent cylinder with screwed-on front and rear disks), the internal batteries, (B) the electronic boards (E), and the heat exchangers (H). The fish were filmed with a video camera (VF) *via* a mirror (M) and the swimming flagellates were filmed with a video microscope (VA). The algal tank had a volume of 1,450 ml and the fish tank had a volume of 1,237 ml.

**Figure 2:**
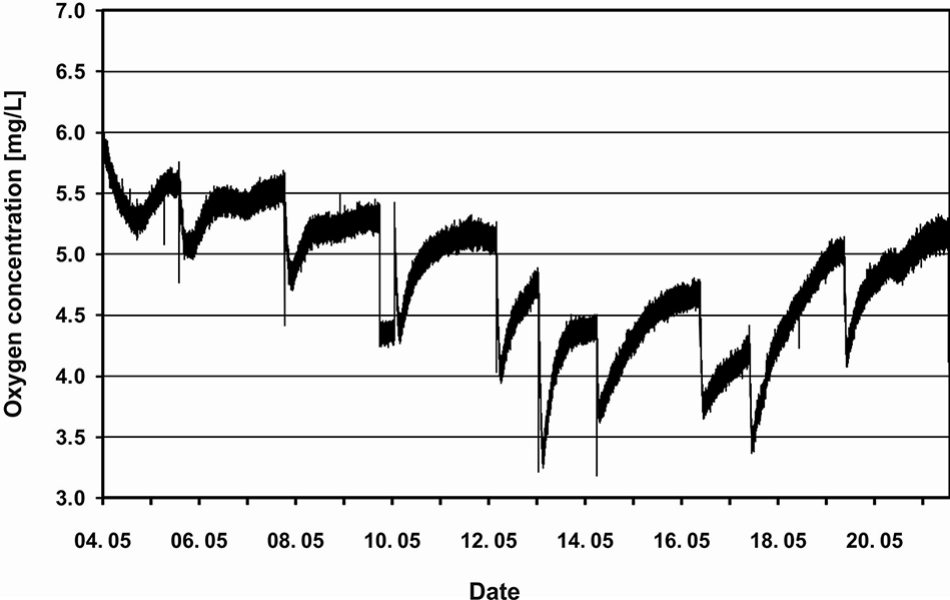
Oxygen concentration (in mg/L) measured in the fish tank of the Aquacells experiment. The sudden drops in the oxygen concentration correspond with the feeding times of the fish. The x-axis shows the dates from May 4 to 22, 2005. (modified from Häder et al., 2006).

The AQUACELLS experiment was mounted in the Russian Foton M1 satellite launched in October 2002. Unfortunately the Soyuz rocket carrying the satellite failed and the hardware was destroyed. A reflight on Foton M2 was successfully launched on 31 May 2005 (Häder et al., 2006). The AQUACELLS hardware was housed in a single container. It consisted of a 1,450-ml tank holding a *Euglena* suspension (**Figure 1**). Photosynthesis was constantly driven by red light-emitting diodes (LEDs). The fish tank had a volume of 1,237 ml and housed 26 larval cichlids (Tilapia, *Oreochromis mossambicus*). Since the fish excrete ammonium the water from the fish tank was first cleaned by a filter with bacteria which metabolized the ammonium to nitrate. Subsequently the water was pumped through 12 cylindrical tubes which span the algal tank to exchange oxygen and carbon dioxide. The oxygen concentration in the fish container was determined with an Oxy 4 min system (Precision Sensing, Regensburg, Germany). Once a day some of the *Euglena* suspension was pumped in a closed loop into a cuvette where the movement of the cells could be observed in infrared with a miniaturized microscope and recorded on video tape. After the mission the tapes were analyzed to determine motility, velocity, swimming direction, and cell form parameters (Häder, 1992; Häder, 2017). The oxygen concentration in the fish tank slowly decreased during the first 9 days and subsequently increased probably due to the fact that 7 fish had died in the mean time (**Figure 2**). The sharp decreases in oxygen coincide with the feeding of the fish when they consume more oxygen due to digestion. After this the concentration increased again.

OMEGAHAB (*Oreochromis Mossambicus-Euglena Gracilis*-Aquatic HABitat) was launched with the Russian Foton-M3 for a 12-day orbital flight mission (Strauch et al., 2008). The algae were contained in a 1,350-ml polycarbonate tank and irradiated with red LEDs to sustain photosynthesis but not to induce phototaxis, which uses a blue-light photoreceptor. For extended space flights within the solar system solar light could be used to drive photosynthesis of the algae. The fish tank had a volume of 1,260 ml and housed 35 larval cichlids. The water from the adjacent fish tank was pumped *via* plastic foam filters, which keep the larvae from leaving the fish tank, through polymer tubes running through the algae container for exchange of O_2_, CO_2_, and ammonium excreted by the fish (Strauch, 2009). Ammonium is a problem in closed life support systems since it is toxic for fish, plants, and humans (Randall and Tsui, 2002; Kudo and Kawashima, 2003; Esteban et al., 2016). *E. gracilis* is a good choice as photosynthetic component since it tolerates ammonium as an N-fertilizer source (Takemura et al., 2017). In addition, it absorbs toxic substances such as nickel and other heavy metals cleaning the water (García-García et al., 2018). However, this could be a disadvantage if the cells are used as food directly or indirectly. The temperature of the fish and *Euglena* media was kept constant using Peltier elements.

**Figure 3:**
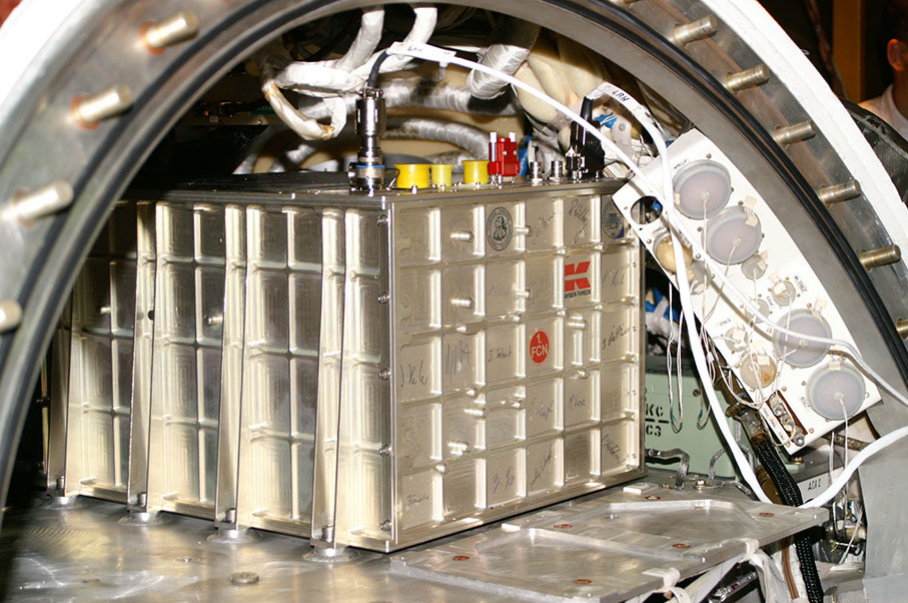
The Omegahab hardware during installation into the Foton satellite. Size: 360 x 400 x 250 mm, weight 18 kg, power consumption 8.1 kWh for the whole mission (Strauch et al., 2008).

In order to monitor motility and orientation, the algal cells were transported by a peristaltic pump into a closed loop containing a chamber where they were recorded for 10 min per day by a custom-made miniaturized microscope on video tape during the mission to monitor their swimming behavior in microgravity. During the initial 6 days the larval fishes lived off their yolk sacks; after that they had to be fed using an automatic feeder. The fish were kept in a 12/12 h day/night cycle (yellow LEDs) and were also filmed to analyze their development and movement. Housekeeping data including temperature were transmitted by downlink and used to adjust the values in an identical ground reference experiment. The experiment container was mounted in the Foton satellite (**Figure 3**) and the Soyuz rocket was launched from Baikonur (Kasachstan). As expected, in the absence of gravity the flagellates swam in random directions and at higher velocities than under 1-g conditions (Häder et al., 2006).

The oxygen content was monitored inside the tank. After an initial increase the oxygen concentration dropped continuously from about 7 to 1 mg/L. Because of the low oxygen level at the end of the mission only 11 of the 26 fishes survived. During fish feeding the value dipped downward.

In cooperation with Chinese scientists a closed aquatic ecosystem of 60 ml was flown. An experiment including two different algae and snails were flown on the Shenzhou 8 mission for more than 2 weeks (Nasir et al., 2014; Li et al., 2017). In addition to testing the O_2_ production and CO_2_ removal several biochemical and molecular genetic experiments have been carried out. The results confirmed that the algae are exposed to stress conditions during microgravity conditions.

Recently the Eu:CROPIS mission has been developed and launched (Hauslage et al., 2018). One new approach is to recycle urine on a nitrifying trickle filter on a lava rock as a fertilizer to grow vegetables (Micro-Tina tomatoes) which are grown from seeds germinated after launch. *Euglena* is used as additional producer of oxygen.

### Outlook

Based on the experience obtained with the various ground-based systems as well as those flown in Earth orbits it seems possible to utilize bioregenerative life support systems on extended manned space missions (Aronowsky, 2017). The next generation of life support systems being developed within the European space life sciences program will concentrate on a modular approach in order to understand the interactions between different species in ecological like support systems (Häder et al., 2018). To optimize the efficacy of such systems the specific needs of each organism has to be explored and the physico-chemical conditions precisely controlled by automatic systems. In case of failure of one or more modules ample backup will be necessary. Careful control of potential contaminants and protection from unpredictable parasites such as viruses, bacteria, and fungi is an essential requirement for future human space travel exploring the solar system and beyond.

## Author Contributions

The author confirms being the sole contributor of this work and has approved it for publication.

## Conflict of Interest

The author declares that the research was conducted in the absence of any commercial or financial relationships that could be construed as a potential conflict of interest.
